# Systematic review and meta-analysis of randomized controlled trials assessing the impact of fish consumption on micronutrient status of children

**DOI:** 10.3389/fnut.2026.1836928

**Published:** 2026-06-09

**Authors:** Molly Ahern, Bahar Kucuk, Maria Wik Markhus, Marian Kjellevold

**Affiliations:** 1 Fisheries and Aquaculture Division, Food and Agriculture Organization of the United Nations (FAO), Rome, Italy; 2 Department of Community Medicine, UiT The Arctic University of Norway, Tromsø, Norway; 3 Department of Seafood, Nutrition and Environmental State, Institute of Marine Research, Bergen, Norway

**Keywords:** animal-source foods, aquatic foods, fish, children, vitamin D, calcium, iodine, iron

## Abstract

**Introduction:**

Fish consumption contributes to overall nutrient intake and health, but its effects on micronutrient status remain uncertain. We conducted a systematic review and meta-analysis to assess the impact of fish consumption on the micronutrient status of children.

**Methods:**

Three databases were searched (April 2025) for studies comparing biomarker-based micronutrient status among children (2–14 years) with fish versus low or no fish intake. Risk of bias was assessed using RoB2, and findings were synthesized through meta- and narrative analyses with GRADE evaluation.

**Results:**

Seven studies (six randomized controlled trials) met inclusion criteria, covering nine biomarkers, including 25(OH)D, urinary iodine concentration, ferritin, hemoglobin, retinol, beta-carotene, calcium, cobalamin, and folate. Three studies had low risk of bias, two moderate, and one high. Meta-analysis was possible only for vitamin D (25(OH)D, 1,074 participants), showing a pooled mean difference of 4.08 nmol/L (95% CI: 1.73–6.43) favoring fish consumption; sensitivity analysis (850 participants) yielded 3.46 nmol/L (95% CI: 1.03–5.65), with no heterogeneity. Results for other biomarkers were largely nonsignificant.

**Conclusion:**

Fish consumption modestly improves vitamin D status in children, though effects may lack clinical relevance. Evidence for other micronutrients is inconsistent, highlighting the need for more rigorous studies.

## 1 Introduction

Over half of preschool children worldwide have micronutrient deficiencies (1) leading to anemia, impaired immunity, impaired brain development, and other health issues (2–5). About 25% of schoolchildren have anemia, often linked to iron deficiency (6); vitamin A deficiency affects 18–20% of adolescent girls in low-income countries (7), and iodine deficiency affected nearly 36.5% of children based on a 2005 study (8), with more recent evidence showing that insufficient iodine intake is widespread globally (9). Vitamin D deficiency is a widespread global health issue, affecting approximately 1 billion people worldwide, with up to 50% of the global population exhibiting insufficient levels, although prevalence varies widely by region and population, with few studies on children and adolescents (10). Global estimates suggest widespread inadequate zinc (17%) and folate (54%) intake (11, 12). Improving children’s nutrition can boost growth, cognition and overall health (13, 14).

Fish^1^ supplies n-3 long-chain eicosapentaenoic acid (EPA) and docosahexaenoic acid (DHA), and essential micronutrients like vitamin A, D, B12, iodine, iron, selenium, zinc (15, 16), and enhances absorption of micronutrients, such as iron and zinc, from plant-source foods when consumed together (17). Due to the EPA and DHA content, fish consumption is linked to cardiovascular and neurodevelopmental benefits (16). Accordingly, organizations like WHO, FAO and EFSA recommend fish as part of a balanced diet during all life stages, including childhood (16, 18). The effect of fish consumption on fatty acid status and associated health outcomes throughout the lifecycle has been well documented in an evidence synthesis comprising systematic review (19), meta-analyses and umbrella reviews (20), as well as expert consultations (16, 21) and risk–benefit assessment (22) with limited or inconclusive evidence for many health outcomes in children.

Food based dietary guidelines (FBDG) often promote fish as a protein-rich food, grouping it with protein-rich menu options, such as animal-source foods or legumes in school meal guidelines and menus (23). Some dietary guidelines emphasize fatty fish (relating to EPA and DHA), while few promote fish as a micronutrient source (24, 25). FBDGs guide programs such as school feeding, aligning school menus with national nutrition guidelines and standards (23, 26). Despite fish supplying over 50% of animal protein in several countries in Africa and Asia (27), it is often excluded from school meals due to food safety and affordability concerns (28). Its impact on micronutrient status remains poorly understood, leading to its undervaluation in nutrition guidelines.

Recent research highlights fish’s potential to reduce micronutrient deficiencies (29–32), supported by data on food composition (15, 30, 33). Previous systematic reviews have highlighted the overall health benefits of fish consumption during the first 1,000 days (from pregnancy to 2 years of age) (34). However, evidence on how fish intake affects micronutrient status—especially in children and adolescents—remains limited. This study aims to systematically review evidence on fish consumption and micronutrient status of children aged 2–14 years, addressing a critical gap between research focused on infants (up until 2 years of age) and women of reproductive age (aged 15–49 years). The findings will inform policy, practice and future research.

## 2 Methods

This systematic review is conducted in accordance with the Cochrane Handbook for Systematic Reviews of Interventions, Version 6.5, 2024 (35) and reported in accordance with the Preferred reporting items for systematic reviews and meta-analyses (PRISMA) 2020 (36) and Synthesis without meta-analysis in systematic reviews (SWiM) (37) checklists (Supplementary material S1).

### 2.1 Criteria for considering studies for this review

The inclusion and exclusion criteria for this systematic review are described below in (S)PICO format (study design, population, intervention, comparison, outcome).

#### 2.1.1 Study design

Randomized controlled trials (RCTs), randomized cross-over trials, non-randomized controlled trials, prospective cohort studies, systematic reviews or umbrella reviews with or without meta-analyses including the study designs mentioned are considered for inclusion for this review. Retrospective cohort studies, case–control studies, cross-sectional studies, animal studies, *in-vitro* studies, systematic reviews or umbrella reviews including only results from the above study types, non-systematic reviews such as scoping reviews or literature reviews, and letters to the editor, opinion pieces, perspective papers were not eligible for inclusion.

#### 2.1.2 Population

This systematic review considered studies with children aged between 2 and 14 years from both sexes. Ages outside of this range were excluded due to metabolic differences and distinct nutritional patterns and/or needs. Studies with populations with chronic conditions were excluded, as well as studies on pregnant women and mother–child pairs with maternal interventions. Nationality/ethnicity is not an exclusion criterion. In the event of mixed populations, this review only considered studies when at least 80% of the population fit the inclusion criteria.

#### 2.1.3 Intervention

The intervention considered for inclusion is fish^2^ consumption. Food-based interventions were our inclusion, hence, interventions with food supplements including fish oil were excluded. In addition, fortified fish products were excluded, such as iron-fortified fish sauces. There were no limitations set for the duration of the intervention. In the case of studies with multiple intervention arms, only the relevant ones for this review were included.

#### 2.1.4 Comparison

For the studies that have comparison groups such as RCTs, studies were included only if the comparison groups were not consuming fish or had lower fish consumption compared to the intervention group. Our aim is to assess the effect of the added fish into diets, rather than comparing different fish type(s) against each other. Hence, studies were excluded if comparison groups differed only in type(s) of fish. In the case of multiple comparison groups, only the relevant ones for this review were included.

#### 2.1.5 Outcome(s)

The outcomes of interest for this review were selected based on established knowledge about nutrients for which fish are good sources. The outcomes of interest are changes in any biomarker of zinc, vitamin A, vitamin B12, iodine, calcium, iron, selenium, vitamin D, or vitamin B9. Studies that investigated environmental contaminants or sustainability issues or studies that estimated intake of specific nutrients from the diet or intake of fish were excluded as they did not give indications about the nutritional status of the consumer.

#### 2.1.6 Other criteria

We only included published journal articles in English language. Non-English language articles, and different publication types such as book chapters, theses, conference abstracts were excluded. Setting, country, year of publication or data collection were not exclusion criterion.

### 2.2 Search strategy

Protocol registries were checked prior to starting the review, to ensure that no ongoing reviews exist on the topic. The search was conducted in three electronic databases that were the most relevant for the topic:

- MEDLINE via Web of Science
- Embase Classic+Embase <1947 to 2025 March 28> via Ovid platform
- Cochrane library

Keywords were structured in (S)PICO format and checked across title abstract and keywords using truncation wild cards and Boolean operators. Terms covered study design (e.g., systematic review RCT prospective cohort); population (e.g., children adolescents); fish and aquatic food [broad terms specific products and selected species relevant to school meal programs (28)] and global production (38) dietary intake (consumption diet) and outcomes (e.g., zinc iron iodine). The reference lists and relevant protocols were screened for additional studies. The last search was done on April 1 2025 and the full search strategy is available in Supplementary material S2.

### 2.3 Study selection and data extraction

Prior to screening, duplicate records were detected by Rayyan and reviewed side-by-side by two researchers before removing any records deemed to be duplicates. Records were screened in Rayyan QCRI by two researchers independently, with disagreements resolved through discussion. Relevant full texts were obtained where possible, and reasons for exclusion recorded. Data from included studies were extracted into an Excel form for this review, covering publication details; study details; sample sizes; population; intervention and comparison characteristics (type, quantity, and form of fish or comparison, timing and duration); outcomes; results; ethics, funding, and conclusions. Study protocols and relevant reports were also reviewed to supplement missing information.

### 2.4 Assessment of methodological quality of included studies

Each RCT was assessed for RoB by two researchers independently using the Cochrane RoB2 tool to assess bias at outcome level (39) across five domains: (1) randomization; (2) deviations from interventions; (3) missing outcome data; (4) outcome measurement; and (5) selective reporting. Bias was rated as low, some concerns, or high for each domain and conflicts were resolved by discussion. Results are visually summarized.

### 2.5 Data synthesis

Meta-analysis was performed only for sufficiently homogeneous studies; otherwise, findings were synthesized narratively. Mean differences (MD) with 95% confidence intervals (CIs) were calculated using a random-effects model and visualized with forest plots. The Hartung-Knapp-Sidik-Jonkman (HKSJ) method was used for minimizing type I error due to limited studies. Heterogeneity was assessed using Tau^2^, Chi^2^, and I^2^ using restricted maximum likelihood estimation (REML).

Sensitivity analyses excluded studies with high RoB, and publication bias was not assessed statistically due to insufficient number of studies (*n* < 10). Missing data were included as reported in the primary studies. Analyses were conducted in Review Manager (40) with an alpha level of 0.05.

### 2.6 Assessment of the certainty of the evidence

Confidence in the meta-analysis findings was evaluated using the Grading of Recommendations, Assessment, Development, and Evaluation (GRADE) approach (41). An adapted version of the GRADE framework was employed to assess evidence quality in the absence of pooled effect estimates (42). The certainty of evidence was assessed using five domains (risk of bias, inconsistency, indirectness, imprecision, and publication bias) and rated as very low, low, moderate, or high certainty. The summary of findings (SoF) table was generated to provide a clear and concise overview of the evidence and its quality.

## 3 Results

### 3.1 Results of the literature search

A total of 1,251 records were identified through database searches. After removing duplicates, 889 articles remained for title and abstract screening. Of these, 25 were reviewed in full text, and ultimately, 6 RCTs for a total of 7 articles were included in this review. No additional studies were found through checking protocols or reference lists.

**Figure 1:**
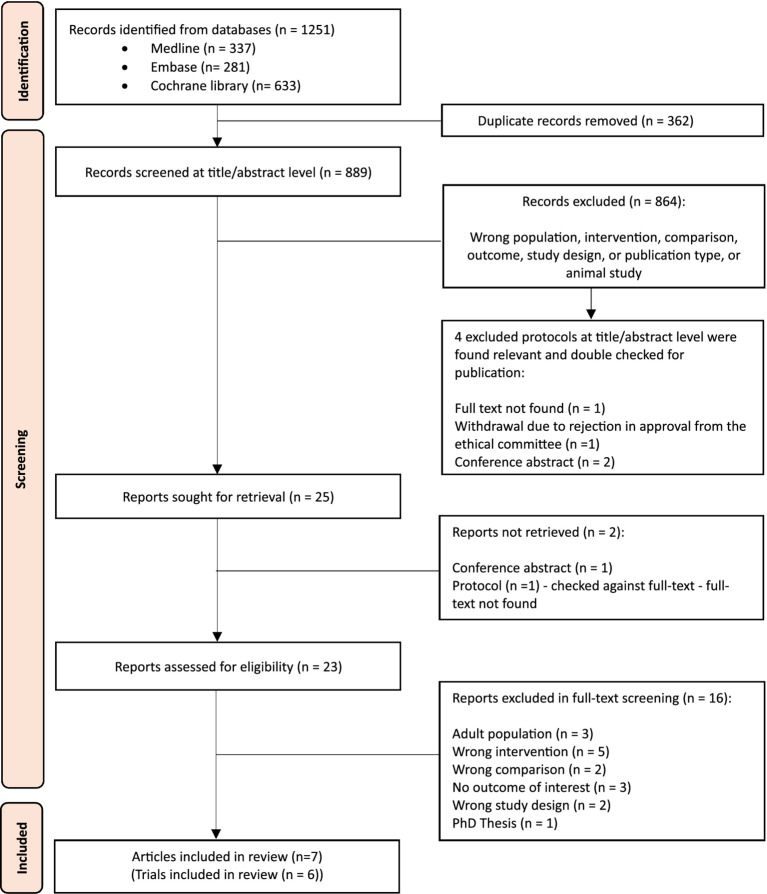
Flow diagram of the study selection process.

The list of studies excluded during full-text screening, along with the reasons for their exclusion, are provided in Supplementary material S3. The flow diagram is shown in Figure 1.

### 3.2 Description of included studies

The included studies (2015–2021) were conducted in Oman (43), Germany (44), Ghana (45), Norway (46–48), and Denmark (49). All studies were conducted in school or kindergarten settings, except one (44) which combined kindergarten and home interventions, and one (49) which used home-prepared meals. Participant age ranged from 4 to 15 years. The FINS-TEENS study (mean age 14.6) was retained as it fit the inclusion criteria (46).

Intervention duration ranged from 12 weeks to 6 months, with fish intake between 150 and 500 g/week. Comparisons included habitual diet, meat/poultry, cheese, or beans. Four studies focused on fatty fish as the intervention (44, 46–48).

Outcomes included s-/p-25(OH)D (vitamin D) (5 studies), UIC (iodine) (3 studies), s-/p-ferritin (iron) (4 studies), p-retinol, p-beta-carotene (vitamin A) (1 study), p-Total calcium (calcium) (1 study), p-hemoglobin (iron) (2 studies), p-cobalamin (vitamin B12) (1 study), and p-folate (vitamin B9) (1 study). The total number of participants ranged from 93 (hemoglobin) to 886 (vitamin D). No studies assessed selenium and zinc. A visual summary in the form of an evidence-and-gap map is provided in Table 1. Urine samples for iodine measurement were collected either at kindergartens or participants’ homes. With one exception (45), all studies collected non-fasting blood samples.

**Table 1:** Evidence-and-gap map for our pre-selected outcomes for inclusion.

Geographic region	Calcium (p-Total calcium)	Iodine (UIC)	Iron (s-/p-ferritin, p-hemoglobin)	Selenium	Vitamin A (p-retinol, p-beta carotene)	Vitamin B9 (p-Folate)	Vitamin B12 (p-cobalamin)	Vitamin D (s-/p-25(OH)D)	Zinc
Africa			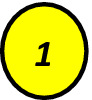						
Asia									
Europe		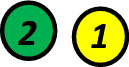	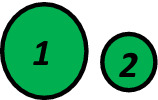			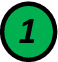	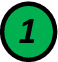	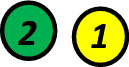	
Middle East	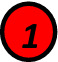				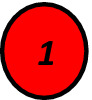			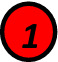	
North America									
Oceania									
South America									

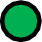
 Color code: low risk of bias; 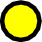 unclear risk of bias/some concerns; 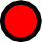 high risk of bias.

Size code: 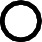 One related biomarker; 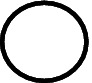 Two related biomarkers.

The number of studies is shown inside each circle.

Detailed study characteristics and dietary composition of the interventions are in Tables 2, 3.

**Table 2:** Characteristics of the included studies.

Study ID	Setting	Data collection period	Population	Intervention/Comparison duration		Outcome(s)	Compliance
				Intervention	Comparison		
Al-Ghannami et al. (43)^a^ Oman RCT	Schools in Muscat Governorate	September 2012 - 2014^b^	*N* = 314 randomized (to three arms), 310 completed Age group: 9–10 years old Girls (n = 174, 56.1%) Boys (*n* = 136, 43.9%) **Exclusion criteria: Children with known hereditary or chronic medical condition which requires medication or suffer from fish or shellfish allergy	12 weeks - Lunch at school		p-25(OH)D (nmol/L) p-Retinol (μmol/L) p-Beta-carotene (μmol/L) p-Total calcium (mmol/L)	Not addressed
				*n* = 108 Fish menu: 100 g lightly grilled fish sandwich with some vegetables (grouper, sea bream, kingfish, emperor, and snapper)	*n* = 116 Habitual food		
Demmelmair et al. (44) Germany RCT	Public and private kindergartens in the area of Munich and homes of the selected participants	March–April 2014	*N* = 205 randomized, 189 completed Age group: 4–6 years old Girls (*n* = 95, 50.3%) Boys (*n* = 94, 49.7%) Test location Kindergarten: (*n* = 150, 79.4%) Home: (*n* = 39, 20.6%) median (IQR) Age (*n* = 185): 5.0 (0.8) Socioeconomic score Mother (*n* = 170): 16.0 (5.0) Father (*n* = 170): 17.0 (5.0) Inclusion criteria: Apparently healthy and an omnivorous diet Exclusion criteria: Any food allergies, severe illness diagnosed by a pediatrician, weight below the 10th percentile or above the 90th percentile, reported dislike of fish, habitual fish intake greater than one meal per week, and use of n-3 supplements during the last three months	16 weeks - Three times a week		s-25(OH)D (nmol/L) UIC (μg/L)	Study diaries of intervention meals show 34.0 (median, IQR 16.9) of 48 study meals (16 weeks times three meals per week) were consumed. No significant difference of the number of consumed meals between the salmon (median 33.6, IQR 20.3) and meat group (34.3, 13.7) was observed (*p* = 0.148).
				*N* = 101 allocated, 96 analyzed Approximately 50 g Atlantic salmon per meal The salmon was filet and vacuum packed in containers of either 200 g salmon burger, 250 g pasta filled with salmon pate, salmon filet in paprika sauce, potato gratin with salmon, or 300 g Bolognese sauce with salmon.	*N* = 104 allocated, 93 analyzed Approximately 50 g meat per meal Meat was vacuum packed in containers of either 200 g beef burger, 250 g turkey filet with pepper sauce, potato gratin with turkey filet, Tortellini with ham filling or 300 g Bolognese sauce with ham.		
				Families could choose among the meals. No side dishes were offered and no recommendations to influence the families’ choice of side dishes were given.			
Egbi et al. (45)^a^ Ghana RCT	Adaklu Kodzobi basic school complex in the Adaklu-Anyigbe district of the Volta Region of Ghana	NI	*N* = 150 randomized (to three arms), 142 completed Age group: 6–12 years Girls (*n* = 73, 48.7%) Boys (*n* = 77, 51.3%) Mean age: 9.1–9.3 years Most (67–71%) of the parents were subsistence farmers, with 55 to 70% of them having attained middle or junior high school education. Inclusion criteria: Parental and individual consent to participate, child not being allergic to cowpea-based foods, not severely anemic (hemoglobin concentration >75 g/L), not on any iron supplements	6 months – Three consecutive weekdays, at lunch		Hemoglobin (g/L) s-Ferritin (ng/mL)	Not addressed
				*N* = 50 allocated, 50 analyzed Fish meal with vitamin C: cowpea-based food containing 3% fish powder served with 33 mg vitamin C/100 mL of vitamin C–rich drink	*N* = 50 allocated, 43 analyzed Vitamin C: cowpea-based food served with 33 mg vitamin C/100 mL of vitamin C–rich drink		
				Each study participant was served 250 g of cowpea-based food otherwise called beans stew plus 20 g of “gari” (roasted fermented cassava dough) locally termed “aborboe” and served 200 mL drink			
Handeland et al. (46)^a^ Norway RCT FINS-TEENS Study	Eight lower secondary schools in Bergen	August 2014–January 2015	*N* = 478 randomized (to three arms), 431 completed Age group: 14–15 years Girls (*n* = 249, 52.1%) Boys (*n* = 229, 47.9%) Mean age with standard deviation: 14.6 ± 0.3 years Most had higher parental education and were non-immigrants, defined as the participant and both of the parents were born in Norway. Income levels of the family were primarily in the middle range, with lower income being the least represented. Inclusion criteria: Knowing the Norwegian language orally and written. Exclusion criteria: Allergy or intolerance to the study foods or supplements	12 weeks - Three times a week, at lunch (11:00–12:00)		s-25(OH)D (nmol/L) s-Ferritin (μg/L) UIC (μg/L)	A mean of 30 ± 6 meals were served to each participant during the intervention. The total intake (dietary compliance) was higher in the supplement group than in the meal groups, and the total intake of meat in the meat group was higher than the total intake of fish in the fish group. The proportion of participants who consumed at least half of the fish/meat/capsules during the trial was 38, 66 and 87% in the fish, meat and supplement group, respectively
				*N* = 159 allocated, 132 analyzed for vitamin D 128 analyzed for ferritin 129 analyzed for iodine Fatty fish: salmon, mackerel and herring	*N* = 160 allocated, 146 analyzed for vitamin D 142 analyzed for ferritin 143 analyzed for iodine Meat/Cheese: chicken, turkey, beef, lamb and cheese		
				In addition, the meals comprised vegetables and/or salad and mainly wholegrain pasta, focaccia, baguette or tortilla and sometimes dressing. Halal meat and gluten free products were provided on request. The meals had a mean weight of 230 g/portion, and the amount of fish/meat was requested to be between 80 and 100 g/portion. Participants were asked not to change any procedures they had besides the intervention, e.g., use of fish-oil supplements or their habitual dietary intake of fish at home.			
Solvik et al. (48) and Øyen et al. (47) Norway RCT FINS-KIDS Study	13 kindergartens in Bergen	December 2014 -February 2015	*N* = 232 randomized, 222 completed Age group: 4–6 years Girls (*n* = 109, 49.1%) Boys (*n* = 113, 50.9%) Mean age with standard deviation: 5.2 ± 0.6 years Mean parental education: 15.4 years Income levels of the family were primarily in the middle range, with higher income being the least represented. Inclusion criteria: Children with sufficient understanding of the Norwegian language to undergo cognitive testing, and whose caregivers had sufficient language skills to answer online questionnaires in Norwegian Exclusion criteria: Any known food allergies.	16 weeks – Three times a week, at lunch		s-25(OH)D_3_ (nmol/L) s-Ferritin (μg/L) UIC (μg/L) p-Cobalamin (pmol/L) p-Folate (nmol/L)	The children consumed a mean (standard deviation) of 2,070 (978) g fish or 2,675 (850) g meat from the study meals (*p* < 0.0001).
				*N* = 114 allocated, 92 analyzed for vitamin D 84 analyzed for ferritin 96 analyzed for iodine 97 analyzed for cobalamin Fatty fish: each meal contained 50–80 g fatty fish (herring/mackerel)	*N* = 118 allocated, 103 analyzed for vitamin D 94 analyzed for ferritin 104 analyzed for iodine 110 analyzed for cobalamin Meat: each meal contained 50–80 g meat (chicken/lamb/beef)		
				A variety of identical side dishes was provided for both groups. Mean weight of each hot lunch meal with standard deviation: 71.1 (10.4)g			
Vuholm et al. (49) Denmark RCT FiSK Junior Trial	Capital Region of Denmark (55° North)	August 2016 – June 2017	*N* = 199 randomized, 197 completed Age group: 8–9 years Girls (*n* = 99, 50.5% [values are rounded]) Boys (*n* = 98, 49.5% [values are rounded]) Median age (interquartile range): 9.6 (0.5) years Approximately 60% of at least one parent of the children were having a minimum of Master’s degree, 20% having bachelor’s degree, and remaining having vocational or short academic degree` or less. Inclusion criteria: Eligible children had to speak Danish, like oily fish and chicken and not consume oily fish more than once per week or take any n-3 LCPUFA supplements 3 months prior to intervention start. Moreover, parents had to read and speak Danish to be properly informed about the study procedures, and for logistic reasons the household should not contain more than five people. Exclusion criteria: Exclusion criteria for the children were serious chronic illnesses and diseases that could interfere with the study outcomes, diagnosed psychiatric illnesses, medication that may affect study outcomes, concomitant participation in other studies involving dietary supplements or blood sampling, and finally only one child from each household could participate in the study. Parents and children received oral information.	12 weeks - Two dinner meals per week and at least three lunch meals per week		s-25(OH)D (nmol/L) Hemoglobin (mmol/L) p-Ferritin (μg/L)	High study food compliance - the median (25th–75th percentile) intake was 375 (325–426) and 400 (359–452) g/week oily fish and poultry, respectively.
		
*N* = 99 allocated, 97 analyzed for vitamin D 96 analyzed for hemoglobin 97 analyzed for ferritin 300 grams per week Fish: Farmed salmon filets to be eaten hot for dinner and could choose from canned mackerel in tomato sauce, marinated herring, smoked trout, smoked mackerel, salmon fish cakes and salmon sausages as cold lunch products.	*N* = 98 allocated, 91 analyzed for vitamin D 91 analyzed for hemoglobin 91 analyzed for ferritin 300 grams per week Poultry: The poultry group received different cuts (whole, breast, thigh and minced) of frozen, organic chicken to be eaten hot for dinner and cold poultry lunch products such as chicken liver pate, poultry sausages, and chicken meat balls.			
		
Parents collected study foods weekly or bi-weekly and received either a fish or chicken recipe booklet as inspiration for preparation of dinner meals with the study foods. The recipes were matched between the poultry and fish group with regard to type of dish and ingredients, as well as energy and fat content. Parents were asked to substitute the provided study products with some of the child’s usually consumed fish, poultry, and red meat and apart from this to maintain the child’s usual dietary and physical activity habits during the intervention.			

^a^Study has multiple arms, only relevant arms for this review were reported. ^b^Information was gathered from the protocol. RCT; Randomized controlled trial, s; serum, p; plasma, UIC; Urinary Iodine Concentration, FINS; Fish intervention studies, FiSK; Fish, children, health, and cognition.

**Table 3:** Composition of the dietary interventions.

Study ID:	Protein g/100 g		Total fat g/100 g		Vitamin D ug/100 g		Iodine ug/100 g		Iron mg/100 g		Mercury mg/kg	
Al-Ghannami et al. (43)	NI		NI		NI		NI		NI		NI	
Demmelmair et al. (44)	Int: 9.87	Comp: 11.33	Int: 9.24	Comp: 5.41	Int: 1.6	Comp: <1.0	Int: 5.4	Comp: 5.0	NI		NI	
Egbi et al. (45)	Int: 21.1	Comp: 18.4	Int: 4.5	Comp: 4.3	NI		NI		Int: 5.4	Comp: 4.1	NI	
Handeland et al. (46)	Int: 10.8	Comp: 11.9	Int: 10.1	Comp: 8.4	Int: 2.1	Comp: <0.1	Int: 5.0	Comp: 2.6	NI		NI	
Solvik et al. (48)^a^ and Øyen et al. (47)	Int: 15.2/13.2	Comp: 15.3	Int: 6.8/24.8	Comp: 13.8	Int: 9.0/2.0	Comp: <1.0	Int: 9.0/21.0	Comp: 5.0	Int: 0.7^b^	Comp: 1.0 ^b^	Int: 0.035/0.017	Comp: <0.2
Vuholm et al. (49)												

All the values are given in means. For the interventions or comparisons having more than one option, average value is reported. ^a^Average means are given for herring/mackerel meals for intervention. ^b^Personal communication, J. Øyen. NI; No Information, Int; Intervention, Comp; Comparison, diff; Difference.

### 3.3 Risk of bias assessment of included studies

Among the six trials (encompassing 7 articles) included in this review, three were assessed as having a low RoB (46–49). Two studies were rated as having moderate RoB (44, 45), while one study was deemed to have a high RoB (43).

Concerns were primarily related to selection bias, performance bias, and reporting bias. These issues arose due to insufficient or missing information on randomization, allocation concealment and baseline differences, lack of blinding, absence of results for some pre-specified measurements, absence of a registered protocol, or unexplained deviations from the protocol.

A detailed breakdown of the RoB assessments is presented in Table 4.

**Table 4:** RoB assessments summary.

Study ID	Selection bias	Performance bias	Attrition bias	Detection bias	Reporting bias	Overall bias
Al-Ghannami et al. (43)	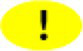	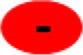	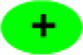	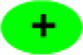	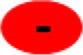	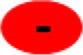
Demmelmair et al. (44)	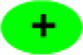	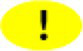	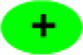	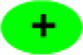	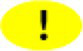	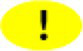
Egbi et al. (45)	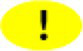	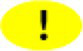	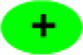	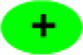	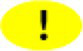	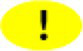
Handeland et al. (46)	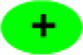	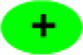	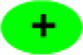	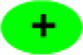	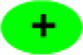	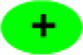
Solvik et al. (48) and Øyen et al. (47)	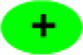	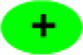	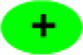	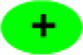	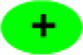	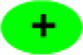
Vuholm et al. (49)	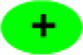	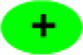	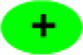	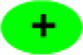	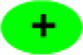	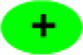

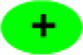
 Low Risk; 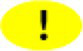 Some concerns; 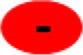 High risk.

### 3.4 Effects of the intervention

**Figure 2:**
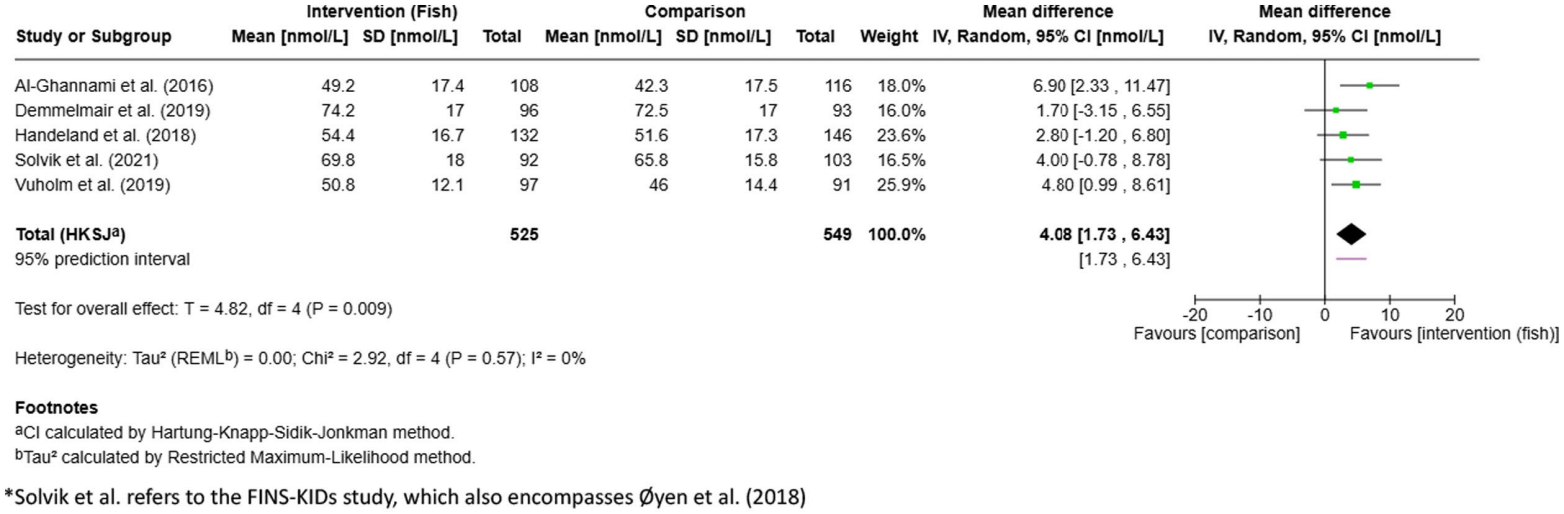
Post-intervention mean difference for vitamin D – pooled estimate (normal/adequate range for s-25(OH)D: >50 nmol/L)I (111). *Solvik et al. refers to the FINS-KIDs study, which also encompasses Øyen et al. (47).

**Figure 3:**
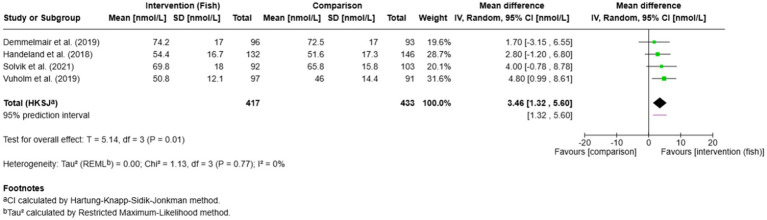
Sensitivity analysis for post-intervention mean difference for vitamin D – pooled estimate (normal/adequate range for s-25(OH)D: >50 nmol/L) (111). *Solvik et al. refers to the FINS-KIDs study, which also encompasses Øyen et al. (47).

**Figure 4:**
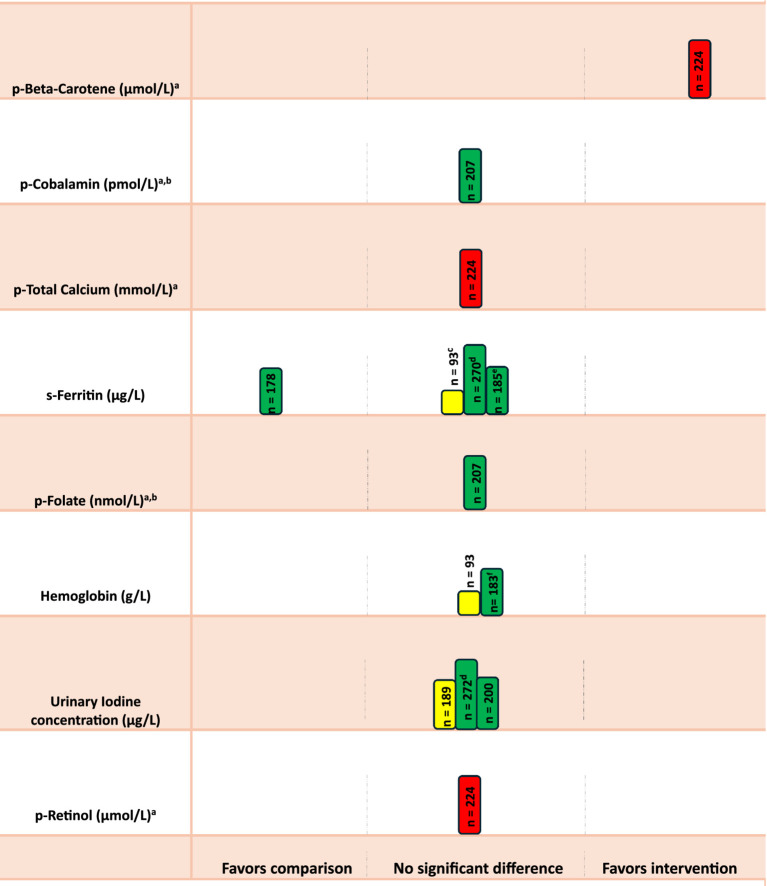
Harvest plot of between group effects for outcomes narratively synthesized. Results are given for mean differences unless specified. Each bar represents one study with the height of the bar being scaled based on the total sample size. The range for total sample size across studies is 93–272. 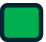 Study has low risk of bias; 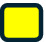 Study has unclear risk of bias/some concerns; 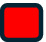 Study has high risk of bias. ^a^Values are for post-intervention difference. ^b^Result is for standardized mean difference. ^c^The unit measured is ng/mL. ^d^Values are adjusted for both baseline and baseline + dietary compliance. ^e^Value is for plasma ferritin. ^f^The unit measured is mmol/L. s; serum, p; plasma.

Due to clinical and methodological heterogeneity across studies, a meta-analysis for only Vitamin D status was conducted with a sensitivity analysis excluding the study by Al-Ghannami et al. (43) due to high RoB. Meta-analyses are presented as forest plots in Figures 2, 3. The remaining outcomes are summarized narratively in Table 5 and visualized in a harvest plot in Figure 4.

**Table 5:** Narrative synthesis of the effect estimates.

Study ID:	Intervention					Comparison					
	N (Change)	Baseline	Post-Intervention	Change	*p* value (within group difference)	N (Change)	Baseline	Post-Intervention	Change	P value (within group difference)	P value (between group difference)
Urinary Iodine Concentration (μg/L) Normal/Adequate Range: UIC 100–299 μg/L (9)											
Demmelmair et al. (44)^a^	96	110 (127.5)	88 (90.5)	NI	*p* = 0.025*	93	110 (118.5)	75 (69)	NI	*p* < 0.001*	*p* = 0.241
Handeland et al. (46)	129	NI	107.7 (72.2)	−11.6 (66.9)	*p* = 0.47	143	NI	125.3 (70.3)	2.4 (73.7)	*p* = 0.70	*p* > 0.05^b^
Solvik et al. (48)/Øyen et al. (47)	96	160.7 (94.6)	143.5 (69.4)	−17.2 (87)	*p* = 0.06	104	151.1 (95.1)	123.6 (62.9)	−27.5 (95.5)	*p* = 0.0041*	*p* = 0.43
s-Ferritin (μg/L) Normal/Adequate Range: >30 μg/L (57)											
Egbi et al. (45)^c^	50	20.7 (18.2;23.6)	27.2 (22.9;32.2)	6.5 (4.7;8.6)	*p* = 0.001*	43	20.1 (16.7;24.4)	23.4 (19.5;28)	3.2 (2.8;3.6)	*p* > 0.05	*p* > 0.05
Handeland et al. (46)	128	NI	39.9 (21.4)	−0.7 (18.1)	*p* = 0.66	142	NI	38.1 (22.7)	−3 (14.8)	*p* = 0.017*	*p* > 0.05^b^
Solvik et al. (48)/Øyen et al. (47)	84	33 (21.6)	26.8 (12.7)	−6.2 (18.2)	*p* = 0.0024*	94	28.3 (16.7)	30.8 (20.3)	2.5 (17.4)	*p* = 0.16	*p* = 0.0013*
Vuholm et al. (49)^a,d^	96	37.4 (18.1)	33.8 (19.4)	−3.5 (14.9)	*p* < 0.05*	89	35.8 (19.9)	37.2 (16.9)	−0.2 (13.5)	*p* > 0.05	*p* = 0.16
p-Retinol (μmol/L) Normal/Adequate Range: >0.7 μmol/L (112)											
Al-Ghannami et al. (43)	108	NI	2.3 (0.8)	NI	NI	116	NI	2.2 (0.7)	NI	NI	*p* > 0.05^e^
p-Beta-carotene (μmol/L) Normal/Adequate Range unknown											
Al-Ghannami et al. (43)	108	NI	1.2 (0.7)	NI	NI	116	NI	0.9 (0.4)	NI	NI	*p* < 0.001^e^ *
p-Total Calcium (mmol/L) Normal Range: 2.2–2.6 mmol/L (111)											
Al-Ghannami et al. (43)	108	NI	2.5 (0.1)	NI	NI	116	NI	2.5 (0.1)	NI	NI	*p* > 0.05^e^
Hemoglobin (g/dL) Normal/Adequate Range: >11 g/dL for children <10 years of age; >12 g/dL for individuals >10 years of age (57)											
Egbi et al. (45)	50	12.01 (0.98)	12.84 (0.72)	0.83 (1.06)	*p* < 0.05*	43	11.91 (1.24)	12.64 (0.89)	0.73 (1.0)	*p* > 0.05	*p* > 0.05
Vuholm et al. (49) ^f^	94	7.8 (0.4)	7.7 (0.4)	−0.1 (0.3)	*p* < 0.05*	89	7.8 (0.4)	7.7 (0.4)	−0.1 (0.3)	*p* < 0.01*	*p* = 0.66
p-Cobalamin (pmol/L)^a^ Normal/Adequate Range:>148 pmol/L (113)											
Solvik et al. (48)	NI^g^	819 (644; 926)	783 (665; 865)	NI	NI	NI^g^	770 (614; 901)	730 (620; 849)	NI	NI	*p* > 0.05^g,h^
p-Folate (nmol/L)^a^ Normal/Adequate Ramge: 6–20 nmol/L (114)											
Solvik et al. (48)	NI^g^	15 (12; 23)	15 (12; 21)	NI	NI	NI^g^	15 (13; 21)	16 (13; 22)	NI	NI	*p* > 0.05^g,h^

Values are given as mean (standard deviation) unless specified. *Statistically significant *p* < 0.05. ^a^Values are given as median (IQR). ^b^Values are adjusted for both baseline and baseline + dietary compliance. ^c^Values are given as geometric means (95%CI) with ng/mL as the unit. ^d^Values are for plasma measurements. ^e^Values are for post-intervention difference. ^f^Values are given as mmol/L as the unit. ^g^*n* = 185 for p-cobalamin, 187 for p folate total baseline, *n* = 207 for total post-intervention. ^h^Values are for standardized mean difference. N; Total number, NI; No Information.

For vitamin D status, the meta-analysis of the post-intervention MD between groups revealed a pooled effect estimate of 4.08 nmol/L (95% CI: 1.73, 6.43) favoring the intervention with no evidence of heterogeneity (Figure 2). Following the sensitivity analysis, the pooled effect estimate decreased to 3.46 nmol/L (95% CI: 1.32, 5.60), again with no heterogeneity detected (Figure 3).

Further, Vuholm et al. (49) found that children who participated in the intervention from September to March in Denmark, avoided the expected winter decline in vitamin D status when consuming fish, while the comparison group showed a significant decline of 11.5 (95% CI: 7.7, 15.2) nmol/L and had a higher prevalence of vitamin D insufficiency. No other studies had such subgroup analyses, although Demmelmair et al. (44) and the FINS-TEENS/KIDS trials (46–48) reported that there was a transition from spring to summer during the intervention.

In addition to unstandardized MD, Solvik et al. (48) reported no significant differences (*p* > 0.05) between the post-intervention means across groups in standardized mean difference (SMD). Further, they adjusted their analyses for baseline measurements, age, gender, and family income and again, no significant difference was observed (SMD: 0.14, 95% CI: −0.06; 0.34, *p* = 0.164).

Per-protocol (PP) analyses were only available in two studies (44, 48), though the latter focused on outcomes not relevant to this review. Solvik et al. (48) defined adherence to the protocol as consuming above the median intake of the study diet. Similarly, their PP analyses showed no significant differences for vitamin D status (SMD: 0.16, 95% CI: −0.20; 0.52, *p* = 0.385).

For the remainder of the outcomes, the difference between-groups was mostly not significant with a few exceptions. For iodine, Solvik et al. (48) found significant increases in standardized (SMD: 0.32, 95% CI: 0.05; 0.59, *p* < 0.05) and adjusted standardized estimates (SMD: 0.28, 95% CI: 0.03; 0.53, *p* = 0.029) favoring intervention group, though PP analyses were not significant (SMD: 0.32, 95% CI: −0.07; 0.71, *p* = 0.107) (48). Additionally, they observed lower post-intervention s-ferritin levels in the fish group than the comparison group and PP analysis, confirming the effect (SMD: −0.30, 95% CI: −0.53; −0.08, *p* = 0.008 and SMD: −0.59, 95% CI: −0.97; −0.19, *p* = 0.003 respectively) (48). The only study that investigated p-beta-carotene reported a significant increase in the intervention group compared to the comparison group for post-intervention MD (Figure 4 and Table 5) (43).

No significant differences were observed between groups for retinol, calcium, hemoglobin, cobalamin or folate (*p* > 0.05). The additional analyses conducted by Solvik et al. (48) on p-cobalamin and p-folate remained non-significant for post-intervention MD across groups (p-cobalamin; adjusted SMD: 0.20, 95% CI: −0.02; 0.43, *p* = 0.081, PP SMD: 0.01, 95% CI: −0.39; 0.41, *p* = 0.971 and p-folate; adjusted SMD: 0.01, 95% CI: −0.18; 0.20, *p* = 0.902, PP SMD: 0.13, 95% CI: −0.23; 0.49, *p* = 0.485) (48).

### 3.5 Certainty of the evidence

Only three outcomes were eligible for GRADE assessment, others appeared in single studies. With all studies being RCTs, assessment started from high certainty and downgraded when needed. The overall certainty of the evidence was rated low for vitamin D (25(OH)D) and UIC, and very low for ferritin. Sensitivity analysis raised the rating for vitamin D to moderate confidence in the evidence. Downgrading was mainly due to inconsistency and imprecision (Table 6).

**Table 6:** Summary of findings.

Outcome	Effect	Duration of intervention	Number of participants (studies)	Certainty of evidence
Meta-analysis				
	Mean difference (95% Confidence Interval)			
Vitamin D (s/p-25(OH)D)nmol/L	4.08 (1.73; 6.43) favoring intervention over comparison group	3–4 months	1,074 (5 RCTs) (43, 44, 46–49)	Low^a,b^ ⊕ ⊕ ⊖⊖
Vitamin D (s-25(OH)D) (nmol/L) – sensitivity analysis	3.46 (1.32; 5.60) favoring intervention over comparison group	3–4 months	850 (4 RCTs) (44, 46–49)	Moderate^b^ ⊕ ⊕ ⊕⊖
Narrative synthesis				
	Mean difference			
s/p-Ferritin (μg/L)	Results were inconsistent with three of the studies showing no significant difference in effect between the groups, while one study having a significant decrease in the intervention group relative to the comparison group	3–6 months	729 (4 RCTs) (45–49)	Very Low^b,c^ ⊕ ⊖ ⊖⊖
Urinary Iodine concentration (μg/L)	Studies showed no significant difference in effect between the groups	3–4 months	661 (3 RCTs) (44, 46–48)	Low^b,d^ ⊕ ⊕ ⊖⊖

Grading of Recommendations, Assessment, Development and Evaluation (GRADE) Working Group grades of evidence (41).

Moderate certainty: We are moderately confident in the effect estimate- The true effect is likely to be close to the estimate of the effect, but there is a possibility that it is substantially different.

Low certainty: Our confidence in the effect estimate is limited- The true effect may be substantially different from the estimate of the effect.

Very low certainty: We have very little confidence in the effect estimate- The true effect is likely to be substantially different from the estimate of effect.

^a^Downgraded by one level due to risk of bias.

^b^Downgraded by one level due to imprecision arising from wide confidence intervals and/or total sample size.

^c^Downgraded by two levels due to inconsistency arising from the differing direction of effect and clinical heterogeneity.

^d^Downgraded by one level due to inconsistency arising from the differing direction of effect.

s; serum, p; plasma.

## 4 Discussion

To our knowledge, this is the first systematic review and meta-analysis examining fish consumption and micronutrient status of children. Among the nine pre-defined outcomes, six studies assessed biomarkers for seven outcomes. Vitamin D status (s-25(OH)D) was the most studied outcome, followed by ferritin and iodine. While individual studies often showed no significant effect, pooled data revealed a significant increase in vitamin D status (s/p-25(OH)D) of 4.08 nmol/L (CI: 1.73; 6.43) favoring fish consumption over the comparison group post-intervention, reduced to 3.46 nmol/L (CI: 1.32; 5.60) in sensitivity analysis, while remaining significant. No statistical heterogeneity was detected; however, the low number of studies limits confidence, and there is low to moderate certainty in the evidence for vitamin D due to RoB and imprecision.

To estimate imprecision, we calculated the review information size (RIS) following Cochrane Handbook and the GRADE Working group (35, 41) (Supplementary material S4). Since there is no rule-of-thumb for the absolute number of participants required for adequate precision in continuous variables, and no well-established minimal clinically important differences (MCID) for our outcomes of interest, we used Cohen’s *d* small effect threshold as the desired effect size, resulting in a RIS of 784 participants. Both analyses met this threshold, but effect sizes were smaller, raising questions about clinical relevance. Nonetheless, in studies where baseline vitamin D levels were close to deficiency (below 50 nmol/L), small increases from fish intake could shift children above sufficiency cut-offs. The mean s-25(OH)D level in two out of five articles included in this review was above this reference value at baseline (44, 48). Among the remaining articles, two of them had a baseline mean of 48.8 nmol/L (46) and 43.1 nmol/L (43) for all children, and one other reported 47.9 nmol/L for the fish group and 48.2 nmol/L for the comparison (49).

When compared to vitamin D supplementation, fish consumption produced modest effects. One serving of fish (50–100 g) containing 32–288 IU vitamin D (Supplementary material S5) corresponded to increases similar to supplementation trials (meta-analysis of supplementation trials resulted in 1.7 nmol/L per 100-IU increment in vitamin D intake) (50), suggesting dietary fish can contribute meaningfully, though not sufficiently, to vitamin D intake.

Our findings are also supported by existing literature. Similar to our findings showing a positive association between fish consumption and vitamin D status (s-25(OH)D), results from a systematic review (34) and observational studies provided evidence that adding fish to maternal and child diets is associated with improved vitamin D (s-25(OH)D) status (51–54). In addition, one study which we excluded from this review found mixed results on the effect of fish consumption on vitamin D status of Danish schoolchildren, as effects differed across seasons and season-modified effects were inconclusive (55). A meta-analysis of RCTs assessing the impact of fish consumption on vitamin D status of adults found that compared with comparison groups, the consumption of fish increased vitamin D (s-25(OH)D) concentrations by 4.4 nmol/L (95% CI: 1.7, 7.1 nmol/L; *p* < 0.0001, *I*^2^ = 25%; 9 studies), with noted differences between fatty fish consumption and lean fish consumption (56).

Regarding iron, we identified studies measuring ferritin and hemoglobin as biomarkers. For ferritin, our narrative synthesis showed mixed results. Some studies showed increased levels within intervention groups from pre- to post-intervention (45), while others showed decreases compared to meat-consuming groups (47, 48). It is worth noting that the comparator group in two studies included here consumed poultry or white meat (46, 49), while others included a mix of red and white meats (chicken, beef or lamb) (47, 48), which may explain some of this variation as red meat is particularly rich in iron. These results may be considered clinically significant. Greater than 30 μg/L (57) is considered adequate. The baseline s-ferritin levels reported in all articles included in this review were near this threshold, with post-intervention mean s-ferritin levels crossing the threshold (to raise ferritin levels beyond the threshold for the fish consuming group (45) and in the comparison group (47, 48)). Although baseline and post-intervention means suggest fish may raise ferritin levels above deficiency thresholds for some children, overall certainty remains very low. Hemoglobin, on the other hand, was assessed in only two studies which found no significant difference across the groups (45, 49). However, overall, due to inconsistency in the results, and the small number of studies and participants, we have very low certainty in the evidence. Our findings are supported by those of other studies assessing the association between fish consumption and iron status (measured by ferritin, soluble transferrin receptor, or hemoglobin). Results are also mixed, for maternal and infant (34, 58–64), adults (65–67) and children (68) in the literature. An RCT conducted in Bangladesh found that consumption of a small fish (*Amblypharyngodon mola*) instead of a larger fish (*Labeo rohita*) had a positive effect on iron status of children (69). Small fish consumed whole are generally more nutrient dense than filet from larger fish, and this could explain this difference.

Iodine results were similarly inconclusive. Three studies identified in this review found no significant effects, and we concluded that we have low confidence in the evidence. Reasons for no effects could be that UIC is a population-level biomarker and that most of the studies used fatty fish in the interventions. In contrast, three other RCTs using lean fish as intervention found positive association with UIC (73–75), while cross-sectional and observational studies show mixed associations (70–72).

We found very limited evidence for the remaining outcomes (p-retinol, p-beta-carotene, p-Total calcium, p-hemoglobin, p-cobalamin, p-folate) and did not assess the certainty of the evidence for these, as it would not be accurate to draw conclusions. For vitamin A, one study reported higher p-beta-carotene but no significant difference in post-intervention means between groups for p-retinol for fish consumers. As beta-carotene comes from plant-source foods, this result is worth acknowledging. However, the study was assessed as having high RoB, primarily due to lack of reporting, and did not include details on the overall diet, thus confounding dietary factors limit conclusions. Evidence from studies with other population groups is mixed. Lartey et al. (59) found that infants that consumed vitamin- and mineral fortified porridge had higher vitamin A status than those that consumed porridge with fish powder (59). In another study, it was not possible to determine the impact of fish consumption on children’s vitamin A status due to lack of a non-fish consuming comparison group (75). Results from a cross-sectional study show that daily fish paste consumption for Thai pregnant women was positively associated with increased concentration of serum retinol and beta-carotene (61).

Our review found only one study that assessed the impact of fish consumption on calcium status (43), vitamin B12 and B9 (48). For calcium, the study included here had high RoB and showed no significant difference detected between intervention and comparison groups. No information was provided about the food served to the comparison/control group in this study other than “habitual diet” (43). A cross-over RCT with isotope marking found that calcium absorption from fish bones was similar to that from supplements (CaCO_3_) (76). Similarly, observational studies suggest associations between fish consumption and calcium status, finding that calcium absorption from meals containing fish (particularly whole fish or fish bones) was comparable to calcium absorption from milk, and improved when compared to powdered limestone (53, 77). Likewise, Consalez et al. (17) suggest that as little as 40 g of fish consumed as part of a meal may enhance absorption of zinc and iron from plant-source foods, although there is insufficient high-quality empirical evidence.

The one study we included (48) which assessed vitamin B12 and B9 showed statistical significance for the difference between groups. Observational studies have found frequent or modest fish consumption is associated with p-vitamin B-12 concentrations for adults (78, 79) and adolescents (80), however dairy and meat products also play a role (81, 82) and results are inconsistent when using different measurements (p-B12, urinary methylmalonic acid) (83). In another RCT, those that consumed an encapsulated fish protein supplement made from salmon by-products had higher serum B12 concentrations than the comparison group (84). We did not identify any other studies that assessed the impact of fish consumption on folate status.

In our review, we found no evidence for the impact of fish consumption on zinc and selenium status. Some links with selenium and zinc have been reported in adults, but child-specific evidence is limited. Two studies found that infants receiving fish-based complementary foods had improved (85) or similar zinc concentrations (86) when compared to legume- or millet-based complementary foods. Other studies have observed elevated zinc levels in populations that consume fish frequently (87). Several studies found positive correlations between fish consumption and plasma selenium, however most were focused on elderly populations (74, 84, 88–91) or pregnant and breastfeeding women (92, 93).

### 4.1 Limitations

This comprehensive review addresses a critical gap by systematically analyzing evidence from RCTs, the highest quality study design among the primary studies in the evidence hierarchy. However, findings should be interpreted cautiously due to heterogeneity, short intervention durations, limited fish species, inconsistent reporting, small sample sizes, and few adjustments for confounders or adherence. Subgroup analyses were not possible.

We were able to pool the effect estimates for only one of our pre-defined outcomes. This was due to both clinical and methodological heterogeneity across the studies, as well as the limited number of studies reporting the outcome of interest. There were substantial differences in the reporting of the outcome data; some studies used different analyses and reporting units due to non-normally distributed data, which limited our ability to pool the results statistically. Moreover, due to the reporting of only post-intervention means across groups, our meta-analyses were conducted based on post-intervention differences, rather than changes from baseline. However, all the studies included in the analyses reported no baseline differences between the groups, with the exception of one study which did not report baseline measurements (43). As this study had high RoB, we excluded it in sensitivity analysis, which supports the robustness of our analyses comparing post-intervention means. In general, outcome measurement was sufficiently similar across studies, except for Egbi et al. (45), which differed from other studies in that they collected non-fasting blood samples. This difference is unlikely to have affected the results, as ferritin reflects iron stores, and is not acutely affected or elevated by food intake.

Information on the participants’ background diet and dietary intake outside of the intervention during the study period was limited and inconsistently reported across studies, thus limiting consideration of confounders and covariates. Reporting on adherence (e.g., number of meals consumed, extent of meal consumption) across the study groups was inconsistent, which could directly influence the results such as the magnitude of effect. The studies’ exclusion criteria did not always explicitly exclude children who take micronutrient supplements habitually, or who have inflammation or other relevant medical conditions. Furthermore, parental education or household income were not considered in all studies although it may have introduced selection bias, as parents with higher socio-economic position may have more control on the child’s habitual diet and adherence to the assigned intervention, particularly those that were conducted at home.

Only the study by Solvik et al. (48) reported PP analysis results for our outcomes of interest, taking adherence into account (48). Both adjustments and PP analyses, in addition to intention-to-treat analyses, are important for understanding the actual intervention effects. For instance, in the case of ferritin, Solvik et al. (48) reported that while the SMD across groups was not statistically significant, this became significant after adjustment and remained significant in PP analysis (48). For iodine, the same study found that SMD and adjusted SMD were significant, although the PP analysis failed to reach statistical significance (48). These findings are important but should also be considered within the limitations of the nature of the interventions, where strict adherence to assigned diets is difficult to enforce. In this context, lack of adherence may reflect a real-world setting.

Due to the small number of studies included, we were unable to perform subgroup analyses (i.e., lean vs. fatty fish; stratification by age group or sex), and we could not statistically assess the publication bias due to the limited number of studies included. Nonetheless, we believe that we adequately captured the available evidence with our comprehensive search strategy, critically appraised the included studies, and reported all the results transparently following the PRISMA and SWiM checklists (Supplementary material S1).

Our findings may be geographically transferable given the diversity of the study populations; however, studies assessing vitamin D status were primarily conducted in northern Europe, where vitamin D deficiency can be a seasonal concern. Included studies are limited in regional representation, as RCTs conducted in Asia tended to focus on the effectiveness of fish-derived supplementation, fortified fish sauce, or adults as the population group (94, 95). This is likely due to challenges with implementing food-based RCTs including fish in the context of low- or middle-income countries, where food safety in fish supply chains remains a challenge (96, 97).

We cannot conclude beyond relatively short-term effects as most interventions included in our review lasted 3–4 months, with only one study extending to 6 months’ duration. Also, given that salmon was the predominant fish species used in the interventions across most studies, the generalizability of the findings to other fish types is constrained. Despite these limitations, this review highlights that fish consumption modestly improves vitamin D status and may contribute to other micronutrients, although stronger, longer, and better-reported studies are needed.

### 4.2 Implications for policy, practice, and further research

Aquatic foods, and more particularly marine fish species, are a unique source of EPA and DHA, as well as one of few natural sources of iodine and vitamin D. Despite the diversity of edible fish species, most interventions in this review used salmon. Given expected growing demands for aquatic food, the EAT-Lancet commission highlighted the need for research assessing the health impacts of consumption of lower-trophic species with lower environmental impact (98).

Recent efforts to aggregate and model food composition data have focused on building evidence of the food composition of diverse aquatic species used for food (99, 100). However, gaps remain for locally important species in LMICs and considering different parts of the fish that are consumed (15). Few studies have assessed fish consumption and nutrient status in these populations; in our review, only one study (45) took place in a LMIC.

Dietary surveys often group aquatic foods with other animal sources (meat, poultry, and fish), while the diversity of other foods such as fruits and vegetables are recognized and divided into several groups based on their nutrient content (i.e., vitamin-A rich fruits and vegetables; dark green leafy vegetables which are rich in non-heam iron) (101). This limits data on species, quantities, and parts consumed (102).

Interventions in this review mainly provided serving sizes of 50–100 g of fish three times per week, consistent with international recommendations (103–105). Few national FBDG mention different serving sizes for children and adolescents, although they are often vague (25). Although fish is one of the few foods that contain vitamin D (106), and serving sizes included in studies within this review were within recommended ranges for children, our study supports previous findings (56) which suggest that fish consumption alone is not sufficient to supply adequate amounts of vitamin D.

The duration of studies included in our review (3–6 months) is sufficient for measuring change in blood parameters and nutritional status, but longer-term studies may provide further evidence on measures that require more time to show an effect (i.e., IQ, anthropometry, neurological changes). The 3–6 month duration of studies included here reflects a real-life scenario in places where fish catch and consumption are seasonal or limited by fisheries management policies, thus affecting fish supply throughout the year (28, 107–109). However, in places where fish availability is stable throughout the year, longer-term studies can provide further evidence.

In countries where children’s fish intake is below recommended amounts, school meal programs offer an entry point for promoting fish consumption, but cultural practices, limited knowledge, low intake and high cost in comparison to school meal program budgets are barriers in many countries where fish consumption by children is low (24, 28).

Recommendations must also consider fish contamination levels and nutrient content, nutritional status of the population, cultural habits and demographics, and local consumption habits (16). There is need to assess the particular risks and benefits for children (110).

## 5 Conclusion

Beyond the recognized contribution of fish to educational and cognitive outcomes for schoolchildren, our meta-analysis provides results that fish consumption can contribute to vitamin D status; however it should be promoted as part of a healthy diet more broadly to maximize micronutrient status of children. One entry point for promoting fish consumption as part of a healthy diet during childhood and adolescence may be through school meal programs. Our narrative review provides evidence of the effect of fish consumption on other micronutrient biomarkers, however evidence is inconsistent and limited. There is a need for more robust studies assessing the effect of fish consumption on micronutrient status of children considering the gaps identified in our review, not only in terms of study design and methodology, but also in the quality and transparency of how findings are reported to allow for future pooled analyses.

## Data Availability

Available datasets from published studies were analyzed in this study. These studies are presented in the study are included in the article/Supplementary material. Analytic code and other materials can be provided on request to the corresponding author.

## Footnotes

1 Here ‘fish’ is used to broadly refer to aquatic animal-sourced foods (i.e., finfish, shellfish, mollusks, etc.)

2 The term “fish” is here defined as finfish (vertebrates) and other seafood (aquatic invertebrates, including crustaceans, mollusks and echinoderms), whether of marine or freshwater origin, farmed or wild. Marine mammals and algae are considered outside the scope.

## Glossary

CI: Confidence Interval
DHA: Docosahexaenoic Acid
EFSA: European Food Safet Authority
EPA: Eicosapentaenoic Acid
FAO: Food and Agricultural Organization of the United Nations
FBDG: Food Based Dietary Guidelines
GRADE: Grading of Recommendations, Assessment, Development, and Evaluation
HKSJ: Hartung-Knapp-Sidik-Jonkman
LC: Long Chain
LMIC: Low- and -Middle Income Countries
MCID: Minimal Clinically Important Difference
MD: Mean Difference
OIS: Optimal Information Size
PP: Per Protocol
PRISMA: Preferred Reporting Items for Systematic Reviews and Meta-Analyses
PUFA: Polyunsaturated Fatty Acid
RCT: Randomized Controlled Trials
REML: Restricted Maximum Likelihood Estimation
RIS: Review Information Size
RoB: Risk of Bias
SD: Standard Deviation
SE: Standard Error
SEP: Socio-economic Position
SMD: Standardized Mean Difference
SoF: Summary of Findings
(S)PICO: Study Design, Population, Intervention, Comparison, Outcome
SWiM: Synthesis Without Meta-Analysis in Systematic Reviews
UIC: Urinary Iodine Concentration
WHO: World Health Organization
