# Supplementary material for: Systematic review and meta-analysis of randomized controlled trials assessing the impact of fish consumption on micronutrient status of children
Source: Front Nutr. 2026 Jun 9;13:1836928. doi: 10.3389/fnut.2026.1836928 (PMC13286829; doi:10.3389/fnut.2026.1836928)
Supplement: Supplementary file 2 [file Table_2.docx]

S2. Full search strategy

|  | **Medline Search String via Web of Science** | **Embase Classic+Embase <1947 to 2025 March 28> Search String via Ovid platform** | **Cochrane Library Search String** |
| --- | --- | --- | --- |
| **1**  **Hits:**  Medline 536,393  Embase 631,187  Cochrane 30,756 | ((((((((((((((((((((((TS=(fish*)) OR TS=(finfish*)) OR TS=(seafood*)) OR TS=("sea food")) OR TS=("blue food")) OR TS=(blue-food*)) OR TS=("aquatic food*")) OR TS=(aquatic-food*)) OR TS=(anchov*)) OR TS=(engraulis)) OR TS=(sardin*)) OR TS=(mackerel*)) OR TS=(pilchard*)) OR TS=(mollusc*)) OR TS=(cod)) OR TS=(tilapia*)) OR TS=(salmon*)) OR TS=(trout*)) OR TS=("fish protein concentrate")) OR TS=("fish protein")) OR TS=("fish powder")) OR TS=("fish flake*")) OR TS=("fish-based product*") | ("fish*" or "finfish*" or "seafood*" or "sea food" or "blue food" or "blue-food*" or "aquatic food*" or "aquatic-food*" or "anchov*" or "engraulis" or "sardin*" or "mackerel*" or "pilchard*" or "mollusc*" or "cod" or "tilapia*" or "salmon*" or "trout*" or "fish protein concentrate" or "fish protein" or "fish powder" or "fish flake*" or "fish-based product*").ti,ab,kw. | fish* or finfish* or seafood* or (sea NEXT food*) or (blue NEXT food*) or (aquatic NEXT food*) or anchov* or engraulis or sardin* or mackerel* or pilchard* or mollusc* or cod or tilapia* or salmon* or trout* or (fish NEXT protein) or (fish NEXT protein NEXT concentrate) or (fish NEXT powder) or (fish NEXT flake) or (fish-based NEXT product) |
| **2**  **Hits:**  Medline 5,126,754  Embase 3,876,164  Cochrane 415,282 | ((((((((((TS=(childhood)) OR TS=(child )) OR TS=(children)) OR TS=("school-age")) OR TS=(schoolchildren)) OR TS=(kid*)) OR TS=(adolescent*)) OR TS=(teen*)) OR TS=(pediatric*)) OR TS=(pre-school*)) OR TS=(preschool*) | ("childhood" or "child " or "children" or "school-age " or "schoolchildren" or "kid*" or "adolescent*" or "teen*" or "pediatric*" or "pre-school*" or "preschool*").ti,ab,kw. | childhood or child or children or school-age or (school NEXT age) or schoolchildren or (school NEXT children) or kid* or adolescent* or teen* or pediatric* or pre-school* or preschool* |
| **3**  **Hits:**  Medline 2,653,428  Embase 3,010,121  Cochrane 2,224,282 | ((((((((((((TS=("systematic review*")) OR TS=("systematic report*")) OR TS=("cochrane reveiw*")) OR TS=("umbrella review*")) OR TS=(meta-analysis)) OR TS=("meta analyses")) OR TS=(RCT*)) OR TS=("randomized controlled")) OR TS=(prospective cohort)) OR TS=(prospective observational)) OR TS=(cohort)) OR TS=(longitudinal)) OR TS=(controlled trial*) | ("systematic review*" or "systematic report*" or "cochrane review*" or "umbrella review*" or "meta-analysis" or "meta analyses" or "RCT*" or "randomized controlled" or "prospective cohort" or "prospective observational" or "cohort" or "longitudinal" or "controlled trial*").ti,ab,kw. | (systematic NEXT review) or (systematic NEXT report) or (cochrane NEXT review) or (umbrella NEXT review) OR meta-analysis OR (meta NEXT analysis) or (prospective NEXT cohort) or RCT or (randomized NEXT controlled) or (prospective NEXT observational) or cohort or longitudinal or (controlled NEXT trial*) |
| **4**  **Hits:**  Medline 1,984,379  Embase 2,270,144  Cochrane 259,352 | ((((((((((((((TS=(eat*)) OR TS=(ate)) OR TS=(intake*)) OR TS=(consumption)) OR TS=(consume*)) OR TS=(consuming)) OR TS=(ingestion)) OR TS=(serving*)) OR TS=(meal*)) OR TS=(diet*)) OR TS=(dine)) OR TS=(dinner*)) OR TS=(lunch*)) OR TS=(breakfast*)) OR TS=(snack*) | ("eat*" or "ate" or "intake*" or "consumption" or "consume*" or "consuming" or "ingestion" or "serving*" or "meal*" or "diet*" or "dine" or "dinner*" or "lunch*" or "breakfast*" or "snack*").ti,ab,kw. | eat* or ate or intake* or consumption or consume* or consuming or ingestion or serving* or meal* or diet* or dine or dinner* or lunch* or breakfast* or snack* |
| **5**  **Hits:**  Medline 2,262,218  Embase 2,084,069  Cochrane 100,482 | ((((((((((((((((TS=(zinc)) OR TS=(zn)) OR TS=("Vitamin A")) OR TS=(Retinol)) OR TS=("Vitamin B12")) OR TS=(B 12)) OR TS=(B12)) OR TS=(Cobalamin*)) OR TS=(Iodine)) OR TS=(Calcium)) OR TS=(Iron)) OR TS=(Selenium)) OR TS=(Vitamin D)) OR TS=(Folic Acid)) OR TS=(Deficiency)) OR TS=(deficient) OR TS=("nutrient status")) | ("zinc" or "zn" or "Vitamin A" or "Retinol" or "Vitamin B12" or "B 12" or "B12" or "Cobalamin*" or "Iodine" or "Calcium" or "Iron" or "Selenium" or "Vitamin D" or "Folic Acid" or "Deficiency" or "deficient" or "nutrient status").ti,ab,kw. | zinc or zn or (vitamin NEXT A) or retinol or (vitamin NEXT B12) or B12 or (B NEXT 12) or cobalamin* or iodine or calcium or iron or selenium or (vitamin NEXT D) or (folic NEXT acid) or deficiency or deficient or (nutrient NEXT status) |
| **1** AND **2** AND **3** AND **4** AND **5** | **Hits: 337** | **Hits: 281** | **Hits: 633** |
| Search Date: 01.04.25 | | | |
