# Supplementary material for: Systematic review and meta-analysis of randomized controlled trials assessing the impact of fish consumption on micronutrient status of children
Source: Front Nutr. 2026 Jun 9;13:1836928. doi: 10.3389/fnut.2026.1836928 (PMC13286829; doi:10.3389/fnut.2026.1836928)
Supplement: Supplementary file 3 [file Table_3.docx]

S3. Excluded studies in full-text screening with reasons of exclusion

| *References (n = 16)* | *Reason for exclusion* |
| --- | --- |
| Al Ghannami S. Nutritional intake, body composition, plasma lipids and fat-soluble vitamins, red blood cell fatty acid profile and behaviour of Omani school children. | Wrong publication type – PhD thesis |
| Andersen AB, Schmidt LK, Faurholt-Jepsen D, Roos N, Friis H, Kongsbak K, et al. The effect of daily consumption of the small fish Amblypharyngodon mola or added vitamin A on iron status: a randomised controlled trial among Bangladeshi children with marginal vitamin A status. Asia Pacific journal of clinical nutrition. 2016;25(3). | Wrong comparison – Article compares different types of fish |
| Charlton KE, Russell J, Gorman E, Hanich Q, Delisle A, Campbell B, et al. Fish, food security and health in Pacific Island countries and territories: a systematic literature review. BMC public health. 2016;16. | Wrong study design - The review does not include any study of the study design of our inclusion criteria |
| da Silva Lopes K, Yamaji N, Rahman MO, Suto M, Takemoto Y, Garcia-Casal MN, et al. Nutrition‐specific interventions for preventing and controlling anaemia throughout the life cycle: an overview of systematic reviews. Cochrane Database of Systematic Reviews. 2021(9). | Wrong intervention – Intervention does not include fish in isolation except for fortified fish products |
| Dalton A, Wolmarans P, Witthuhn RC, van Stuijvenberg ME, Swanevelder SA, Smuts CM. A randomised control trial in schoolchildren showed improvement in cognitive function after consuming a bread spread, containing fish flour from a marine source. Prostaglandins, leukotrienes, and essential fatty acids. 2009;80(2). | No outcome of interest |
| Gibson RS, Yeudall F, Drost N, Mtitimuni BM, Cullinan TR. Experiences of a community-based dietary intervention to enhance micronutrient adequacy of diets low in animal source foods and high in phytate: a case study in rural Malawian children. 2003;133(11):3992S‐9S. | Wrong intervention – Intervention does not include fish in isolation. They have other components included that might affect the interpretation of the results |
| Kongsbak K, Thilsted SH, Wahed MA. Effect of consumption of the nutrient-dense, freshwater small fish Amblypharyngodon mola on biochemical indicators of vitamin A status in Bangladeshi children: a randomised, controlled study of efficacy. 2008;99(3):581‐97. | Wrong comparison – Article compares different types of fish |
| La Banudi LB, Leksono P, Anasiru MA. Effect of Fish-Based Diet on Malnourished Children: A Systematic Review. Iranian journal of medical sciences. 2024;49(3). | Wrong intervention |
| Masset E, Haddad L, Cornelius A, Isaza-Castro J. Effectiveness of agricultural interventions that aim to improve nutritional status of children: systematic review. BMJ (Clinical research ed). 2012;344. | Wrong intervention – No fish intervention in the form of consumption |
| Narchi H, Kochiyil J, Al Hamad S, Yasin J, Laleye L, Al Dhaheri A. Hypovitaminosis D in adolescent females - An analytical cohort study in the United Arab Emirates. Paediatrics and International Child Health. 2015;35(1):36 EP - 43. | Wrong study design – Cross-sectional |
| Navas-Carretero S, Pérez-Granados AM, Sarriá B, Carbajal A, Pedrosa MM, Roe MA, et al. Oily fish increases iron bioavailability of a phytate rich meal in young iron deficient women. 2008;27(1):96‐101. | Wrong population - Adults |
| Navas-Carretero S, Pérez-Granados AM, Schoppen S, Sarria B, Carbajal A, Vaquero MP. Iron status biomarkers in iron deficient women consuming oily fish versus red meat diet. 2009;65(2):165‐74. | Wrong population - Adults |
| Petersen RA, Damsgaard CT, Dalskov S-M, Sorensen LB, Hjorth MF, Andersen R, et al. Effects of school meals with weekly fish servings on vitamin D status in Danish children: secondary outcomes from the OPUS (Optimal well-being, development and health for Danish children through a healthy New Nordic Diet) School Meal Study. Journal of nutritional science. 2015;4. | Wrong intervention - The intervention itself was not fish directly. In their previous report, they found out that the intervention group had a higher intake of fish. Hence, the results of this study could be seen as comparing high fish group to low, however, they also found out that some other food groups are high in intake compared to control too. So, it is hard to say that the effect is coming from fish |
| Roberts M, Tolar-Peterson T, Reynolds A, Wall C, Reeder N, Rico Mendez G. The Effects of Nutritional Interventions on the Cognitive Development of Preschool-Age Children: A Systematic Review. Nutrients. 2022;14(3):532. | No outcome of interest |
| Robinson MF, Rea HM, Friend GM, Stewart RD, Snow PC, Thomson CD. On supplementing the selenium intake of New Zealanders. 2. Prolonged metabolic experiments with daily supplements of selenomethionine, selenite and fish. 1978;39(3):589‐600. | Wrong population - Adults |
| Skotheim S, Dahl L, Handeland K, Froyland L, Lie O, Oyen J, et al. Design of the FINS-TEENS study: A randomized controlled trial assessing the impact of fatty fish on cognitive performance in adolescents. Scandinavian journal of public health. 2017;45(6). | No outcome of interest – Only the intake/composition of the diet was measured |
