# Supplementary material for: Systematic review and meta-analysis of randomized controlled trials assessing the impact of fish consumption on micronutrient status of children
Source: Front Nutr. 2026 Jun 9;13:1836928. doi: 10.3389/fnut.2026.1836928 (PMC13286829; doi:10.3389/fnut.2026.1836928)
Supplement: Supplementary file 4 [file Table_4.docx]

**S4. Review Information Size Calculation and Comparison of Pooled Effect Estimates with Cohen’s d for Minimal Clinically Important Difference**

**As there is an absence of a pre-specified desired effect size for our outcomes related to this specific review question, we adopted Cohen’s small effect threshold of 0.2 as the benchmark for the minimal clinically important difference (MCID). Accordingly, and in line with Cochrane and GRADE recommendations, the optimal information size (OIS) for continuous outcomes was calculated using the formula:**

**N_total_ = (((Z_α/2_​ + Z_β_​)^2^ x 2 x σ^2^) / Δ^2^) x 2 where:**

**N = number of participants required to detect a meaningful difference**

**Z_1-α/2_​: Critical value for the desired significance level, here taken as 1.96 for alpha level 0.05**

**Z_1-β_​: Critical value for the desired power where beta is type 2 error rate, here taken as 0.84 for 80% power**

**σ^2:^ Pooled variance of the outcome**

**Δ: Minimal clinically important difference / desired effect size**

**For both main meta-analysis and sensitivity analysis: N = (((1.96 + 0.84)^2^ x 2) / 0.2^2^) x 2 = 784**

**Since no statistical heterogeneity was observed (I^2^ = 0); *the Review Information Size (RIS) equals the Optimal Information Size (OIS), as shown by:***

**RIS=OIS x (1/ (1−I^2^))​**

**RIS = OIS x (1/(1-0)) = OIS**

**We further evaluated the MCID by comparing it to our pooled effect estimates expressed as Cohen’s d, calculated as:**

**d= (​x̅_1_​− x̅_2_) / ​​Standard Deviation (SD)_pooled_**

**SD = Standard Error (SE) x √n​ (total sample size) where:**

**SE = (Upper confidence limit − Lower confidence limit) / (2 x Z_1-α/2_)**

**Effect sizes calculated for the main meta-analysis:**

**SE_pooled_ = (6.43 – 1.73) / (2 x 1.96) ≈ 1.2**

**SD_pooled_ = 1.2 x √1074 ≈ 39.3**

**4.08 / 39.3 ≈ 0.1 < 0.2**

**Effect sizes calculated for the sensitivity analysis:**

**SE_pooled_ = (5.60 – 1.32) / (2 x 1.96) ≈ 1.1**

**SD_pooled_ = 1.1 x √850 ≈ 32.1**

**3.46 / 32.1 ≈ 0.1 < 0.2**

***Reference: Guyatt GH, Oxman AD, Kunz R, Brozek J, Alonso-Coello P, Rind D, Devereaux PJ, Montori VM, Freyschuss B, Vist G, Jaeschke R. GRADE guidelines 6. Rating the quality of evidence—imprecision. Journal of Clinical Epidemiology. 2011 Dec 1;64(12):1283-93.***

***Corrigendum: Guyatt G, Oxman AD, Kunz R, Woodcock J, Brozek J, Helfand M, et al. Corrigendum to GRADE guidelines 6. Rating the quality of evidence—imprecision. J Clin Epidemiol. 2021;137:265.***
