# Supplementary material for: Systematic review and meta-analysis of randomized controlled trials assessing the impact of fish consumption on micronutrient status of children
Source: Front Nutr. 2026 Jun 9;13:1836928. doi: 10.3389/fnut.2026.1836928 (PMC13286829; doi:10.3389/fnut.2026.1836928)
Supplement: Supplementary file 5 [file Table_5.docx]

**S5. Estimates of Vitamin D IU / serving of fish in studies where food composition data was included**

| **Vitamin D** | **mcg / 100g in the fish** | **Serving Size** | **Mcg/serving** | **IU / serving [1mcg =40 IU]** |
| --- | --- | --- | --- | --- |
| **Demmelmair** | **1.6** | **50g** | **0.8** | **32 IU** |
| **Handeland** | **2.1** | **80-100g** | **1.68-2.1** | **67.2-84 IU** |
| **Solvik** | **9.0 (mackerel)**  **2.0 (herring)** | **50-80g** | **4.5-7.2**  **1.0-1.6** | **180-288 IU**  **40-64 IU** |
